# An assessor-blinded, randomized comparative trial of transcutaneous auricular vagus nerve stimulation (taVNS) combined with cranial electroacupuncture vs. citalopram for depression with chronic pain

**DOI:** 10.3389/fpsyt.2022.902450

**Published:** 2022-08-04

**Authors:** Shaoyuan Li, Zixuan Zhang, Yue Jiao, Guixing Jin, Yue Wu, Fengquan Xu, Yufeng Zhao, Hongxiao Jia, Zongshi Qin, Zhangjin Zhang, Peijing Rong

**Affiliations:** ^1^Institute of Acupuncture and Moxibustion, China Academy of Chinese Medical Sciences, Beijing, China; ^2^Psychiatry Department, The First Hospital of Hebei Medical University, Shijiazhuang, China; ^3^Psychological Department, Guang'anmen Hospital, China Academy of Chinese Medical Sciences, Beijing, China; ^4^Data Centre of Traditional Chinese Medicine, China Academy of Chinese Medical Sciences, Beijing, China; ^5^Psychiatry Department, Beijing Anding Hospital, Capital Medical University, Beijing, China; ^6^The School of Chinese Medicine, The University of Hong Kong, Pokfulam, Hong Kong SAR, China

**Keywords:** taVNS, acupuncture, depression, chronic pain, randomized clinical trial

## Abstract

**Background:**

Depression accompanying chronic pain (CP) is one of the most common comorbid psychiatric disorders. This study aimed to investigate the effectiveness of transcutaneous auricular vagus nerve stimulation (taVNS) combined with electroacupuncture at Baihui (GV20) and Yintang (GV29) acupoints compared with citalopram.

**Methods:**

Sixty patients with depression and pain comorbidity were enrolled in a prospective 8-week, single-blind, randomized controlled trial. Participants were randomly assigned to receive either taVNS combined with electroacupuncture treatment (taVNS: 8 weeks, 3 sessions per week; electroacupuncture: 8 weeks, twice per day, no drugs) or citalopram treatment (8 weeks, 40 mg/day). The primary outcome was Montgomery–Åsberg Depression Rating Scale (MADRS). The secondary endpoints were evaluated using the McGill Pain Questionnaire (SF-MPQ), self-reported 36-Item Short Form Survey (SF-36), Pittsburgh Sleep Quality Index (PSQI), Hamilton Depression Rating Scale (HAMD) and Hamilton Anxiety Scale (HAMA).

**Results:**

Both the taVNS combined with electroacupuncture and citalopram groups had significant reductions in depressive and pain symptoms, as indicated by the decrease in MARDS and SF-MPQ scores. Regarding the analgesic effect, the pain intensity score of the SF-MPQ showed a larger reduction with citalopram than with taVNS combined with electroacupuncture at 6 weeks (*P* = 0.036). The reduction in the BP score of the SF-36 was higher at week 4 (*P* = 0.000), with no significant difference observed at week 8 (*P* = 0.1110). This result indicated that the pain intensity can be improved rapidly with citalopram compared with taVNS combined with electroacupuncture. Similarly, the comparison of PSQI scores at 4, 6, and 8 weeks indicates that there was no significant difference between groups, except in the use of sleeping medications. At week 6, higher medication use was found in the citalopram group than in the taVNS combined with electroacupuncture group (*P* = 0.049).

**Conclusion:**

In summary, compared with citalopram, taVNS combined with electroacupuncture produces similar positive effects on depressive and pain symptoms in patients with depression and chronic pain, which last for at least 8 weeks.

## Introduction

Chronic pain (CP) is a highly prevalent and disabling illness that is costly and poses a substantial burden on patients, their significant others, and society. The prevalence of CP in the United States was reported to be 30.7%, meaning that approximately 1 in 3 Americans lives with CP (1). CP has high comorbidity with depression, with a mean prevalence rate of comorbid major depressive disorder (MDD) in patients with CP ranging from 18% in population-based settings up to 85% in specialized pain clinics (2). In patients with depression, the prevalence of pain ranges from 15 to 100% (mean prevalence, 65%) (3). Although it is generally understood that pain and depression are common comorbidities and that their combination is costlier and more disabling than either condition alone, their interaction is not fully understood. Pain and depression share potential genetic factors (4). Genes and stress interact to alter neuronal size and function in depression. Neurotransmitters such as glutamate, substance P, serotonin, norepinephrine, dopamine, BDNF, and gamma-aminobutyric acid are activated in CP (5). These same neurotransmitters, especially serotonin and norepinephrine, have long been considered key to understanding depression (6).

CP and concomitant depression is common and can recur easily, but prolonged medication use carries risks of addiction, tolerance and obvious toxic side effects (7–10). Transcutaneous auricular vagus nerve stimulation (taVNS) is a non-invasive electrical stimulation method that overcomes potential barriers to the use of vagus nerve stimulation (VNS) by stimulating the afferent fibers of the vagus nerve in the ear to produce an effect similar to classical VNS in reducing depressive symptoms (11). Acupuncture has been recommended by the World Health Organization for the treatment of CP and has even been approved by the U.S. military and Veterans Administration (12). It has been proven that acupuncture and transcutaneous electric stimulation have a lasting analgesic effect (12–15). The underlying mechanism of its therapeutic effects is mainly through the periphery, spinal cord and spinal cord at the central level (16–18). Additionally, evidence-based medicine has verified that acupuncture has the advantages of being simple, safe and effective for anxiety and depressive mood disorders (19, 20), with the mechanism of relevance among neuroendocrine function, cell signal transduction, hippocampal neurogenesis and proinflammatory cytokines (17, 21). However, as a number of these studies lack methodological vigor, further appropriately designed clinical studies are recommended to verify their conclusions. Despite evidence suggesting that acupuncture has therapeutic benefits for patients with CP and comorbid depression, the relative benefits of acupuncture compared to commonly used antidepressants remain unknown. Citalopram, one of the first selective serotonin reuptake inhibitors (SSRIs) introduced to the market, is a widely used treatment for depression (16, 22, 23). The aim of this assessor-blinded, randomized clinical trial is to evaluate the effectiveness of taVNS combined with electroacupuncture treatment compared with citalopram for 8 weeks in patients with CP and mild to moderate comorbid depression. We hypothesize that taVNS combined with electroacupuncture will be superior to citalopram, as measured by the Montgomery–Åsberg Depression Rating Scale (MADRS), Simplify the McGill Pain Questionnaire (SF-MPQ), self-reported 36-Item Short Form Survey (SF-36), Pittsburgh Sleep Quality Index (PSQI), Hamilton Depression Rating Scale (HAMD) and Hamilton Anxiety Scale (HAMA).

## Methods

### Patient selection

The multicentre study recruited patients from The First Hospital of Hebei Medical University, Guang'anmen Hospital of CACMS, and Beijing Anding Hospital of Capital Medical University *via* advertisements, posters, flyers or physician referrals. Patients were enrolled from the outpatient departments of the three hospitals above from May 12, 2018, to April 15, 2019.

The inclusion criteria were as follows: (1) patients with at least one psychogenic pain event and a visual analog scale (VAS) score of 3 or more; (2) patients meeting the Diagnostic and Statistical Manual of Mental Disorders (DSM-5) diagnostic standard [Depressive symptoms typically last at least 2 weeks; Changes in appetite and weight, sleep disorders, and psychomotor agitation or hysteresis; Lack of energy; Feelings of worthlessness or guilt; Difficulty thinking, concentrating, or making decisions (23, 24)]; (3) patients aged 18 to 50 years old; patients with first-onset depression; (4]) patients with mild-to-moderate depression who scored 12–30 points on the MADRS; (5)volunteer participants who were willing to cooperate and adhere to the treatment.

The exclusion criteria were as follows: (1) pregnant women. (2) patients with serious suicidal thoughts or suicidal behavior. (3) patients with other severe organic diseases, such as malignant tumors and liver failure. (4) patients with an ongoing addiction to drugs and alcohol. (5) patients with a history of schizophrenia or other mental disorders. (6) patients with cognitive impairment or personality disorders. After passing a pre-screening performed by study physicians in accordance with the inclusion and exclusion criteria, potentially eligible patients provided informed consent to participate in the presence of a study physician.

### Study design

A randomized, 8-week, assessor-blind controlled clinical trial was developed, consisting of taVNS combined with electroacupuncture and citalopram groups. The protocol was approved by the Institutional Ethics Committee of the China Academy of Chinese Medical Sciences (CACMS) (2017-07-31-2). All participants were informed about the study and had to provide written informed consent. This study has been registered at Clinicaltrials.gov (NCT03282110).

### Randomization

The population comprised 60 patients diagnosed with CP and comorbid depression. Eligible patients were randomly assigned in a 1:1 ratio to the taVNS combined with electroacupuncture or citalopram group *via* a central randomization system. The randomization sequence was generated in blocks of varying size and stratified by the center. The definition of the block size and the factor were 4 and center, respectively, used in stratification. Information on each participant's group allocation was sealed in opaque envelopes by an independent researcher who was not involved in outcome assessments at each clinical site.

### Sample size calculation

According to the formula, $n={\left(Z_{\alpha }+Z_{\beta }\right)}^{2}\times (1+1/k)\times p$ (1-p)/ (p1-p2)^2^ (25), α = 0.05, β = 0.1, k = 1, p1 = 0.50, p2 = 0.10, p = 0.3. p = (p1+kp2)/ (1+k)=0.3. The sample size is Calculated as follows: *n* = (1.64+1.28)^2^× 2 × 0.3 × (1–0.3)/[(0.5–0.1)^2^], N is 22.38, or about 23 cases, that is, the sample size of a single group is 23 cases, so a total of 46 cases are required for this experiment. Take into account the rate of loss. The total of cases is 60.

### Outcomes

The primary outcome was evaluated with the MADRS (26), which has been validated and used in clinical studies including people with and without pain. Lower scores of MADRS indicated the better mental state. The effective rate was defined as the number of participants with a 50% or greater reduction from baseline in the MADRS at weeks 4 and 8. The secondary outcomes were evaluated with SF-MPQ (27), which is widely used to assess the pain condition, the lower the scores of SF-MPQ, the better the mental state of the patients. Other secondary endpoints were evaluated with SF-36 (28) [including Physical Functioning (PF), Role-Physical (RP), Bodily Pain (BP), General Health (GH), Vitality (VT), Social Functioning (SF), Role-Emotional (RE), Mental Health (MH) and Health Transition (HT)], PSQI, HAMD and HAMA. The higher the SF-36 score, the better the health, the lower the scores of PSQI, HAMD and HAMA, the better the mental state of the patients. All endpoints were measured at weeks 0, 4, and 8 by trained interviewers who were blinded to treatment information.

### Interventions

#### taVNS combined with electroacupuncture group

##### taVNS

Patients took a seated position or laid on their sides. After skin disinfection, electrodes were attached to the auricular concha to be stimulated (Hwato brand SDZ-IIB electronic stimulator, Suzhou Medical Application Factory, Suzhou, China), which is similar to our previous clinical studies. The stimulation parameters included the following: 1) a dilatational wave of 4/20 Hz was used (4 Hz for 5 s, 20 Hz for 10 s, the sparse and dense wave alternated). 2) the intensity of stimulation adjusted individually based on the tolerance of the patient. 3) each treatment lasted for 30 min, 2 times per day (once in the morning, the other in the evening), for 5 consecutive days from Monday to Friday per week for 8 weeks. After group randomization, taVNS group patients were trained on how to use the taVNS device so that it could be self-administered at home. The stimulated region is located at the auricular conch, where there is a rich vagus nerve branch distribution.

##### Electroacupuncture

**Figure 1:**
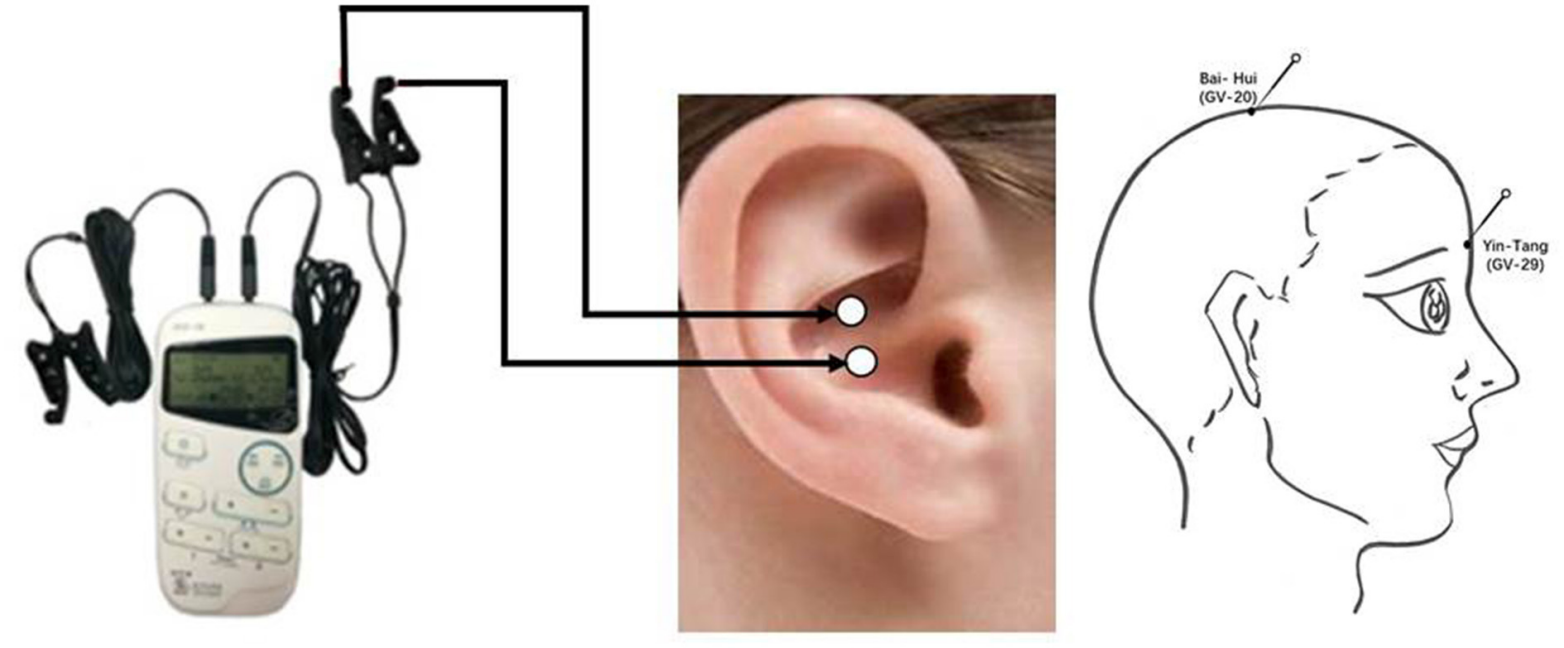
Location of acupuncture and taVNS. taVNS, transcutaneous auricular vagus nerve stimulation.

The electroacupuncture acupoints were Baihui (GV20) and Yintang (GV29), which are located in the parietal and frontal skin that distributes the supraorbital nerve of the trigeminal nerve (Figure 1). Electroacupuncture was conducted for 3 sessions, Monday to Friday every other day, per week over 8 consecutive weeks. Disposable acupuncture needles (0.30 mm in diameter and 25–40 mm in length) were inserted obliquely at a depth of 10–30 mm into the two acupoints, through which electrical stimulation with continuous waves with 2 Hz at 9 volts was delivered with an electrical acupuncture simulation instrument (Hwarto, SMY-10A). The use of a low frequency rather than high frequency was adopted because it could induce biochemical changes in the brain in a more favorable manner for alleviating depressive symptoms. The intensities of stimulation were adjusted to a level at which subjects felt most comfortable. Each simulation lasted 30 min, with 3 sessions per week over 8 consecutive weeks.

#### Citalopram group

Patients in the citalopram group received oral citalopram. Citalopram was provided at an initial dose of 10 mg daily for 3 days, followed by 20 mg daily for 4 days. A daily dose of 40 mg of citalopram, which was taken for 7 weeks, was provided after 1 week. At the end of the study (8 weeks), most of the patients in the citalopram group continued treatment under the guidance of the study physicians.

### Statistical analysis

A descriptive analysis was used, with continuous variables expressed as the mean ± sd and categorical variables expressed as frequencies and percentages. We used *t-test* for continuous outcomes and *Chi-square test* or *Fisher's exact test* for binary outcomes to compare the differences between the groups. The changes from baseline of the primary outcome were analyzed by fitting linear mixed-effects models with repeated measures analysis using the baseline as a covariate, treatment, visit, and their interaction as fixed effects, the intercept as the random effect. The same analysis was used for other longitudinal continuous variables. Besides, effect size was showed by calculating the Cohen's d value, and >0.8 was considered large effect. All statistical analyses were performed using SAS version 9.4 (SAS Institute) or R version 4.1.2. *P* < 0.05 indicated a statistically significant difference.

## Results

### Participants and baseline characteristics

**Figure 2:**
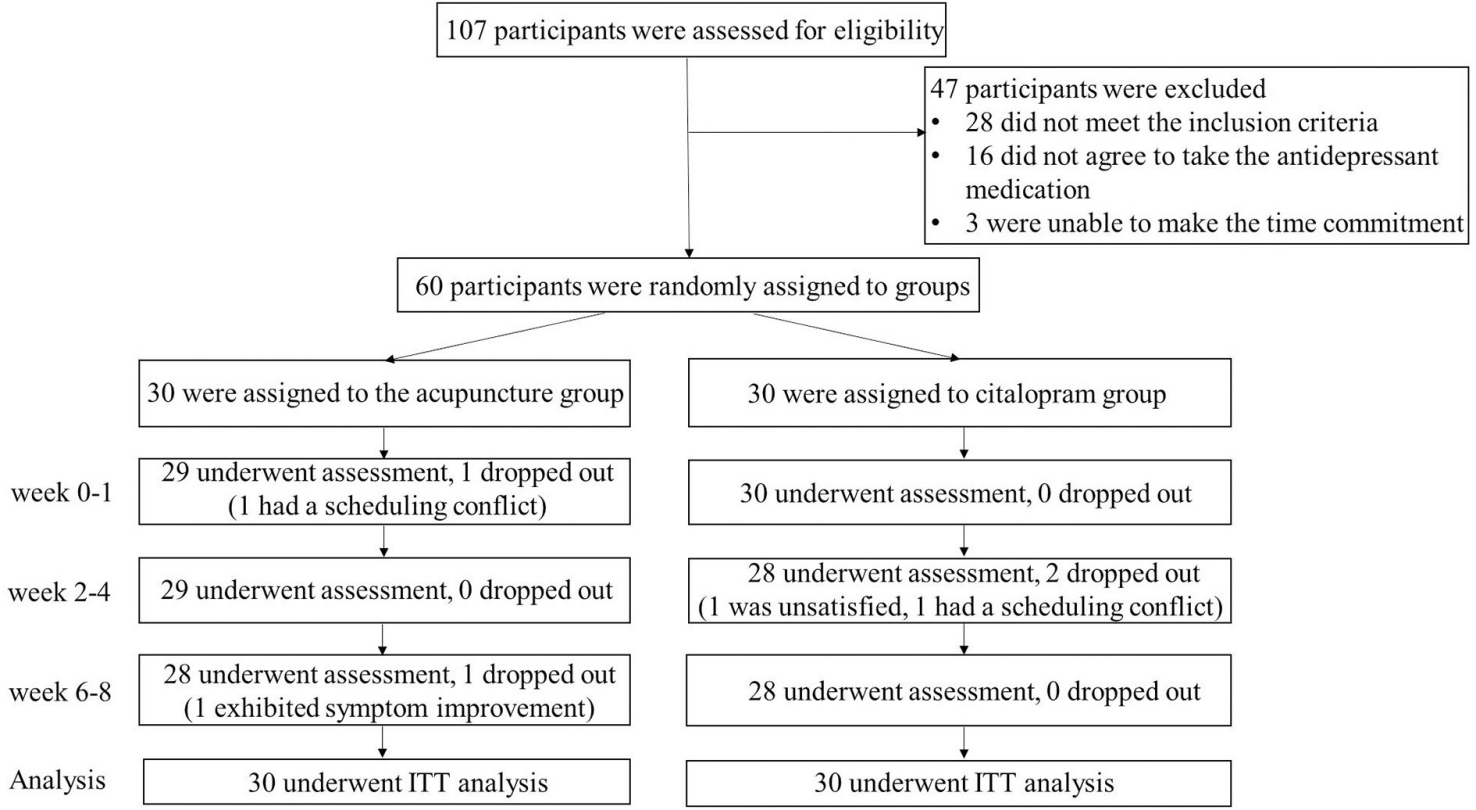
CONSORT chart. ITT, Intention-to-treat.

Initially, of the 107 patients screened, 60 participants were enrolled and randomly divided into two groups (30 in the taVNS combined with electroacupuncture group and 30 in the citalopram group). Some patients were excluded from the study for the following reasons: reluctance to be assigned randomly to a treatment group, refusal of antidepressant medication, and inability to be assessed every 2 weeks. At week eight (end of treatment), 28 subjects in the taVNS combined with electroacupuncture group and 28 subjects in the citalopram group had completed the study. Specifically, two patients dropped out of the taVNS combined with electroacupuncture group (one due to scheduling conflicts at week one and one due to significant improvement at week eight); two patients dropped out of the citalopram group (one due to the lack of a satisfactory effect and one due to scheduling conflicts) (Figure 2).

The baseline characteristics were comparable between the two groups (Table 1), except for systolic blood pressure, which was lower in the taVNS combined with electroacupuncture group. The study population suffered from a wide variety of pain conditions, including chronic back pain, migraines, neuropathic pain, osteoarthritis and fibromyalgia.

**Table 1:** Demographic and clinical characteristics of the patients at baseline.

**Characteristics**	**taVNS combined with electroacupuncture (*N* = 30)**	**Citalopram** **(*N* = 30)**	***P*-value**
Age (yr)—Mean (SD)	34.77 (8.59)	39.40 (8.05)	0.035
Sex—n/N (%)			>0.999
Male	9 (30.00)	8 (26.67)	
Female	21 (70.00)	22 (73.33)	
Systolic blood pressure (mm Hg)—Mean (SD)	124.03 (14.09)	132.70 (10.56)	0.009
Diastolic blood pressure (mm Hg)—Mean (SD)	81.60 (12.47)	83.97 (7.90)	0.384
Heart rate—Mean (SD)	78.10 (9.69)	75.30 (8.82)	0.247
MADRS—Mean (SD)	21.20 (5.37)	23.83 (5.43)	0.064
SF-MPQ—Mean (SD)			
Sensory	4.50 (2.16)	5.07 (2.33)	0.333
Affective	5.50 (2.87)	5.37 (3.00)	0.861
VAS	5.78 (1.60)	6.43 (1.64)	0.125
Pain intensity	2.70 (0.65)	3.10 (0.88)	0.051
Total	18.48 (5.71)	19.97 (5.76)	0.321
SF-36—Mean (SD)			
PF	80.90 (16.47)	77.17 (18.79)	0.417
RP	25.83 (33.79)	19.17 (29.13)	0.416
BP	53.50 (16.47)	45.77 (17.70)	0.085
GH	35.47 (22.89)	30.43 (21.57)	0.384
VT	38.83 (21.28)	35.67 (25.52)	0.604
SF	58.33 (20.59)	47.08 (22.43)	0.048
RE	27.78 (32.85)	17.78 (31.24)	0.232
MH	36.67 (18.44)	33.07 (18.07)	0.448
HT	41.67 (31.03)	35.83 (26.82)	0.439
PSQI—Mean (SD)			
Total score	11.57 (3.34)	13.07 (3.71)	0.105
Subjective sleep quality	1.87 (0.68)	2.33 (0.71)	0.012
Sleep latency	2.17 (0.91)	2.27 (0.83)	0.658
Sleep duration	1.93 (1.01)	2.07 (1.08)	0.624
Habitual sleep efficiency	1.40 (1.22)	1.63 (1.27)	0.471
Sleep disturbance	1.27 (0.52)	1.50 (0.57)	0.104
Sleeping medications	0.47 (1.07)	0.67 (1.18)	0.496
Daytime dysfunction	2.50 (0.73)	2.67 (0.76)	0.390
HAMD—Mean (SD)	18.33 (4.05)	19.53 (3.32)	0.215
HAMA—Mean (SD)	17.23 (5.08)	19.03 (5.82)	0.207

### Comparison between taVNS combined with electroacupuncture and citalopram on primary and secondary outcome measurements

The adjusted scores for the MARDS, SF-MPQ, SF-36, PSQI, HAMD and HAMA at different time points are illustrated in Table 2. Between group differences in the effective rate were −4.06% (95%CI: −17.75%, 25.86%) and 7.14% (95%CI: −17.15%, 30.30%, each *P* > 0.05) at weeks 4 and 8. Both the taVNS combined with electroacupuncture and citalopram groups showed reductions in depressive and pain symptoms, as indicated by the decrease in MARDS and SF-MPQ scores at week 8. We found that during the 8-week treatment, the antidepressant effect of taVNS combined with electroacupuncture remained similar to that of citalopram in terms of the MADRS, HAMD and HAMA scores, with differences between groups of −0.47 (95%CI: −2.33, 1.39), −0.64 (95%CI: −2.26, 0.98) and 0.44 (95%CI: −1.48, 2.36), respectively (*P* > 0.05).

**Table 2:** Changes in baseline and endpoint data on outcome measures for the intent-to-treat sample.

**Outcomes**	**Time**	**taVNS combined with electroacupuncture (*N* = 29)**	**Citalopram** **(*N* = 30)**	**Adjusted Difference (95% CI)**	***P*-value**	**Effect Size**
MADRS (95%CI)	4 week	−7.92 (−9.21, −8.76)	−8.35 (−9.65, −7.04)	0.43 (−1.42, 2.28)	0.649	0.65
	8 week	−12.96 (−14.27, −1.93)	−12.49 (−13.79, −11.19)	−0.47 (−2.33, 1.39)	0.619	−0.71
Effective rate (%)	4 week	4/29 (13.79)	5/28 (17.86)	4.06 (−17.75, 25.86)	0.730	–
	8 week	19/28 (67.86)	17/28 (60.71)	7.14 (−17.15, 30.32)	0.780	–
SF–MPQ (95%CI)						
Sensory	4 week	−1.49 (−2.03, −0.96)	−1.72 (−2.26, −1.17)	0.22 (−0.54, 0.98)	0.564	0.84
	8 week	−3.05 (−3.60, −2.50)	−3.04 (−3.59, −2.49)	−0.01 (−0.79, 0.77)	0.978	−0.04
Affective	4 week	−2.83 (−3.49, −2.17)	−2.76 (−3.42, −2.09)	−0.07 (−1.01, 0.87)	0.883	−0.21
	8 week	−3.86 (−4.53, −3.18)	−3.91 (−4.59, −3.24)	0.05 (−0.90, 1.01)	0.914	0.15
VAS	4 week	−1.58 (−2.09, −1.08)	−1.99 (−2.50, −1.48)	0.41 (−0.31, 1.12)	0.266	1.60
	8 week	−3.26 (−3.77, −2.74)	−3.38 (−3.89, −2.87)	0.12 (−0.61, 0.85)	0.741	0.46
Pain intensity	4 week	0.13 (−0.22, 0.48)	−0.46 (−0.82, −0.10)	0.59 (0.08, 1.09)	0.023	3.28
	8 week	−0.95 (−1.32, −0.59)	−1.21 (−1.57, −0.85)	0.26 (−0.26, 0.77)	0.328	1.41
Total	4 week	−5.72 (−7.12, −4.33)	−6.98 (−8.40, −5.58)	1.27 (−0.72, 3.25)	0.210	1.77
	8 week	−11.03 (−12.46, −9.63)	−11.64 (−13.07, −10.21)	0.61 (−1.41, 2.63)	0.553	0.84
SF−36 (95%CI)						
PF	4 week	8.66 (5.17, 12.15)	5.83 (2.28, 9.39)	2.83 (−2.16, 7.82)	0.261	1.61
	8 week	11.91 (8.37, 15.44)	11.8 (8.24, 15.35)	0.11 (−4.91, 5.13)	0.965	0.06
RP	4 week	17.98 (7.16, 28.81)	6.39 (−4.63, 17.41)	11.59 (−3.87, 27.05)	0.139	2.13
	8 week	41.52 (30.55, 52.49)	30.5 (19.48, 41.51)	11.02 (−4.54, 26.59)	0.161	2.01
BP	4 week	12.11 (7.34, 16.88)	14.12 (9.26, 18.97)	−2.01 (−8.86, 4.85)	0.559	−0.84
	8 week	21.86 (17.03, 26.70)	24.26 (19.41, 29.11)	−2.39 (−9.30, 4.51)	0.490	−0.99
GH	4 week	14.32 (8.76, 19.87)	6.49 (0.84, 12.14)	7.83 (−0.11, 15.77)	0.053	2.80
	8 week	19.36 (13.74, 24.99)	18.24 (12.59, 23.89)	1.12 (−6.87, 9.12)	0.779	0.40
VT	4 week	17.34 (11.58, 23.10)	8.87 (3.00, 14.73)	8.47 (0.25, 16.70)	0.044	2.92
	8 week	25.49 (19.67, 31.30)	21.54 (15.68, 27.41)	3.94 (−4.32, 12.21)	0.343	1.36
SF	4 week	13.70 (7.72, 19.68)	7.96 (1.87, 14.05)	5.74 (−2.88, 14.37)	0.187	1.91
	8 week	22.59 (16.51, 28.67)	20.90 (14.81, 27.00)	1.69 (−7.02, 10.39)	0.699	0.56
RE	4 week	14.53 (2.15, 26.92)	10.05 (−2.56, 22.65)	4.48 (−13.22, 22.19)	0.861	0.72
	8 week	26.34 (13.76, 38.93)	27.90 (15.30, 40.51)	−1.56(−19.41, 16.28)	0.614	−0.25
MH	4 week	14.23 (9.63, 18.83)	8.09 (3.40, 12.77)	6.14 (−0.45, 12.72)	0.067	2.65
	8 week	20.14 (15.49, 24.79)	16.8 (12.12, 21.49)	3.34 (−3.29, 9.96)	0.317	1.43
HT	4 week	9.30 (2.01, 16.59)	3.69 (−3.73, 11.11)	5.61 (−4.81, 16.02)	0.285	1.53
	8 week	7.38 (0.02, 14.74)	7.26 (−0.16, 14.68)	0.12 (−10.35, 10.59)	0.982	0.03
PSQI (95%CI)						
Total score	4 week	−3.03 (−4.11, −1.94)	−2.33 (−3.42, −1.23)	−0.70 (−2.25, 0.86)	0.377	−1.26
	6 week	−4.39 (−5.49, −3.29)	−3.40 (−4.50, −2.30)	−1.00 (−2.56, 0.57)	0.211	−1.78
	8 week	−5.47 (−6.57, −4.37)	−4.40 (−5.50, −3.30)	−1.07 (−2.63, 0.50)	0.180	−1.92
Subjective sleep quality	4 week	−0.70 (−0.95, −0.46)	−0.64 (−0.88, −0.39)	−0.07 (−0.42, 0.28)	0.699	−0.49
	6 week	−1.00 (−1.24, −0.76)	−0.81 (−1.06, −0.57)	−0.19 (−0.54, 0.16)	0.295	−1.53
	8 week	−1.07 (−1.32, −0.83)	−0.99 (−1.24, −0.75)	−0.08 (−0.43, 0.27)	0.657	−0.64
Sleep latency	4 week	−0.58 (−0.86, −0.31)	−0.62 (−0.90, −0.34)	0.04 (−0.35, 0.43)	0.841	0.29
	6 week	−0.87 (−1.15, −0.59)	−0.91 (−1.18, −0.63)	0.04 (−0.36, 0.43)	0.855	0.28
	8 week	−1.16 (−1.43, −0.88)	−0.98 (−1.26, −0.70)	−0.18 (−0.57, 0.21)	0.371	−1.28
Sleep duration	4 week	−0.49 (−0.77, −0.20)	−0.52 (−0.81, −0.23)	0.03 (−0.37, 0.44)	0.868	0.21
	6 week	−0.83 (−1.12, −0.54)	−0.56 (−0.84, −0.27)	−0.28 (−0.68, 0.13)	0.184	−1.85
	8 week	−0.87 (−1.16, −0.58)	−0.77 (−1.06, −0.48)	−0.10 (−0.50, 0.31)	0.640	−0.68
Habitual sleep efficiency	4 week	−0.57 (−0.93, −0.20)	−0.57 (−0.94, −0.20)	0 (−0.52, 0.52)	0.991	0.00
	6 week	−0.74 (−1.11, −0.38)	−0.57 (−0.94, −0.20)	−0.18 (−0.70, 0.35)	0.507	−0.91
	8 week	−1.07 (−1.44, −0.70)	−0.71 (−1.08, −0.34)	−0.35 (−0.88, 0.17)	0.182	−1.92
Sleep disturbance	4 week	−0.29 (−0.44, −0.14)	−0.14 (−0.29, 0.01)	−0.15 (−0.36, 0.06)	0.172	−2.00
	6 week	−0.40 (−0.54, −0.25)	−0.29 (−0.43, −0.14)	−0.11 (−0.32, 0.10)	0.311	−1.45
	8 week	−0.32 (−0.47, −0.17)	−0.25 (−0.40, −0.10)	−0.07 (−0.29, 0.14)	0.495	−0.92
Sleeping medications	4 week	0.11 (−0.28, 0.50)	0.65 (0.25, 1.04)	−0.53 (−1.09, 0.02)	0.060	−2.70
	6 week	−0.03 (−0.43, 0.36)	0.65 (0.25, 1.04)	−0.68 (−1.24, −0.12)	0.018	−3.38
	8 week	−0.03 (−0.43, 0.36)	0.47 (0.07, 0.86)	−0.50 (−1.06, 0.06)	0.080	−2.49
Daytime dysfunction	4 week	−0.63 (−0.86, −0.39)	−0.46 (−0.70, −0.22)	−0.16 (−0.50, 0.17)	0.343	−1.40
	6 week	−0.71 (−0.95, −0.47)	−0.75 (−0.99, −0.51)	0.03 (−0.31, 0.37)	0.842	0.33
	8 week	−1.07 (−1.31, −0.83)	−1.00 (−1.24, −0.76)	−0.07 (−0.41, 0.27)	0.673	−0.57
HAMD (95%CI)	4 week	−7.43 (−8.56, −6.30)	−7.27 (−8.41, −6.13)	−0.16 (−1.78, 1.45)	0.841	−0.27
	8 week	−11.66 (−12.80, −10.51)	−11.02 (−12.16, −9.88)	−0.64 (−2.26, 0.98)	0.437	−1.10
HAMA (95%CI)	4 week	−7.20 (−8.55, −5.84)	−7.32 (−8.66, −5.97)	0.12 (−1.80, 2.04)	0.903	0.17
	8 week	−10.48 (−11.84, −9.13)	−10.92 (−12.27, −9.58)	0.44 (−1.48, 2.36)	0.652	0.64

To explore the effects of the two different treatments on the quality of life, we measured the SF-36 score with 9 items, including PF, RP, BP, GH, VT, SF, RE, MH and HT, at weeks 4 and 8. No significant difference (*P* > 0.05) was found in terms of changes to the SF-36 score at any assessment time point (Table 2).

Similarly, the comparison of PSQI scores at 4, 6, and 8 weeks indicates that there was no significant difference between the two groups, except in the use of sleeping medications. At week 6, higher medication use was found in the citalopram group [adjusted score and 95% CI: 0.65 (0.25, 1.04)] than in the taVNS combined with electroacupuncture group [adjusted score and 95% CI: −0.03 (−0.43, 0.36), *P* = 0.018].

### Safety and compliance

Based on the patients' treatment journals and verbal reports, the main side effect of acupuncture was sharp pain during stimulation (one subject). For citalopram, side effects included digestive problems such as nausea, sour regurgitation (two subjects), early wake-up, drowsiness (one subject) and dry eyes (one subject).

## Discussion

For nearly a century, the relevant theory and application research of the ear has greatly promoted the development of auricular acupoint therapy in traditional Chinese medicine, and finally proposed the concept of taVNS. taVNS can transmit signals to the solitary tract nucleus by activating the vagal afferent fiber, and then regulate the body function, and taVNS has been widely used in the clinical treatment of epilepsy, depression, consciousness disorders and other neurological diseases (29). Our randomized controlled trial with 107 depression patients failed to show a significant advantage of taVNS over citalopram (30). Therefore, we expect to involve additional invasive intervention to enhance the effectiveness of taVNS in the treatment of depression combined with CP.

Acupuncture on GV20 and GV29, those forehead acupoints are found innervated by the trigeminal nerve, while the stimulation process activates the sensory pathways of the trigeminal nerve (24). This study found that taVNS combined with electroacupuncture treatment results in symptom improvement, reflected in the MADRS, SF-MPQ, SF-36, PSQI, HAMD and HAMA scores, and that this improvement is similar to that of citalopram, a commonly prescribed anti-depressant for patients with pain and comorbid mild to moderate depression. This result is consistent with the findings of several meta-analyses assessing the effectiveness of acupuncture monotherapy in comparison with antidepressants (31, 32). Although we found that participants in the taVNS combined with electroacupuncture group showed a lower analgesic benefit at week four compared with the citalopram group, no significant difference was found at week eight, indicating that citalopram may have a faster analgesic effect than acupuncture. taVNS combined with electroacupuncture has a certain protective effect on the maintenance of patients' energy, suggesting that this method is safer than citalopram group. No serious adverse events related to the interventions were reported. Our results demonstrated the potential of taVNS and electroacupuncture for the simultaneous treatment of mild and moderate MDD with CP comorbidity.

Regarding clinical research, effectiveness of taVNS and electroacupuncture on Baihui (GV20) and Yintang (GV29) in relieving depressive and pain symptoms has been implied by previous studies. Hein and colleagues (33) found that 2 weeks of taVNS treatment could significantly reduce Becker Depression Index scores when compared to the sham condition. Nevertheless, there was no significant difference in the HAMD scores. In a subsequent non-randomized clinical study (11), we found that after the fourth week, the taVNS group had greater decreases in the 24-item HAMD score. The clinical improvements continued until week 12. In another recent study (34), Trevizol and colleagues found that the 17-item HAMD scores were significantly reduced after 2 weeks of taVNS treatment in a single-arm study. Liu CY and colleagues (35) found that patients in the acupuncture (GV20 and GV29) group showed a lower HAMD score compared with the sham acupuncture group. Acupuncture has been increasingly used as an integrative or complementary therapy for chronic pain, which is well tolerated with little risk of serious adverse effects (36, 37). In an open randomized trial, transcutaneous electrical nerve stimulation, infrared laser therapy and acupuncture on GV20 combined with other acupoints were proven to be effective in reducing the frequency of headache attacks (38). Liu CZ and colleagues carried out a multicentre randomized sham-controlled trial of patients with knee osteoarthritis and found that compared to SA, intensive acupuncture resulted in less pain and better function at week 8, and these effects persisted until week 26 (39, 40). Acupuncture is well tolerated with little risk of serious adverse effects on pain syndromes (36).

Peijing Rong and colleagues used CUMS rats and chronic constriction injury (CCI) of the sciatic nerve to generate a depression and chronic somatic pain comorbidity model. The results showed that electroacupuncture and taVNS have antidepressant and analgesic effects (20). The results of this trial are consistent with the findings reported in the abovementioned studies, demonstrating the benefit of taVNS and electroacupuncture in relieving symptoms of MDD and CP.

Citalopram is a second-generation antidepressant that has been approved by regulatory agencies and shows good acceptability (41). Studies have found that it is more efficacious than placebo in adults with MDD. It has been reported that citalopram is also used as an antidepressant for chronic non-cancer pain in children and adolescents. We found that the therapeutic effects from taVNS and electroacupuncture were similar to those from citalopram in patients with MDD and CP. In addition, participants in the taVNS combined with electroacupuncture group showed a lower analgesic benefit at week six than those in the citalopram group. This finding also suggests that citalopram may have a faster analgesic effect than taVNS and electroacupuncture. Future studies are needed to validate this finding.

We did not detect serious side effects for taVNS based on the patients' treatment journals and verbal reports. One subject reported sharp pain during stimulation, which may be due to hypersensitivity of the stimulation area. For citalopram, the side effects reported include digestive problems such as nausea, sour regurgitation, waking up early, drowsiness, and dry eyes. This result is consistent with the literature on the side effects of citalopram (41).

It is the first time to investigate the effectiveness of combination therapy on depression with CP. There are still several limitations of this small sample exploratory study that should be noted. First, this study was assessor-blinded; the participants could not be blinded due the nature of the treatment. Second, taVNS combined with electroacupuncture was administered for 8 weeks. Thus, our results only demonstrate the similarity between the effects of taVNS combined with electroacupuncture and those of citalopram over 2 months. Third, we included only patients with mild to moderate depression and comorbid CP. We are not sure if the effects of taVNS combined with electroacupuncture and citalopram are similar for patients with severe depression or other symptoms. We also did not exclude patients based on prior treatment; thus, patients included in this study may have some heterogeneity. In addition, some of the patients may have had residual symptoms of a more severe episode (refractory), and some could have had a persistent depressive disorder. We are not sure if the effects of taVNS combined with electroacupuncture and citalopram are similar for treatment-resistant depression, which is the target population of classic VNS. Finally, taVNS and citalopram were administered by the patients themselves, so we cannot be completely sure that all patients precisely followed the experimental protocol. Nevertheless, the self-administration of taVNS provides direct evidence of the feasibility of the widespread application of this method, which could significantly reduce treatment expenses.

## Conclusion

In summary, we found that compared to citalopram, a commonly prescribed anti-depression agent, taVNS combined with electroacupuncture produces similar effects in relieving depressive and pain symptoms in patients with MDD and CP for at least 8 weeks. Therefore, this safe and low-cost peripheral neuromodulation approach may be considered a therapeutic option for the multidisciplinary management of patients with depression and comorbid CP.

## Data availability statement

The original contributions presented in the study are included in the article/supplementary material, further inquiries can be directed to the corresponding author.

## Ethics statement

The studies involving human participants were reviewed and approved by the Institutional Ethics Committee of the China Academy of Chinese Medical Sciences (CACMS) (2017-07-31-2). The patients/participants provided their written informed consent to participate in this study.

## Author contributions

SL: conceptualization, project administration, formal analysis, writing—original draft, writing—review and editing, and funding acquisition. ZiZ: project administration, data section, writing—original draft, and writing—review and editing. YJ: data section and methodology. GJ, YW, HJ, and FX: data section. YZ: methodology and formal analysis. ZQ and ZhZ: methodology and writing—review and editing. PR: funding acquisition, conceptualization, project administration, supervision, and writing—review and editing. All authors contributed to the article and approved the submitted version.

## Funding

The work was supported by Young Elite Scientists Sponsorship Program by CAST (2021-2023ZGZJXH-QNRC001), National Natural Science Foundation of China (82004181), Excellent Youth Fund of Chinese Academy of Chinese Medicine (ZZ15-YQ-048), and International Cooperation Project of Traditional Chinese Medicine in CACMS (GH2017-07).

## Conflict of interest

The authors declare that the research was conducted in the absence of any commercial or financial relationships that could be construed as a potential conflict of interest. The reviewer LH declared a shared affiliation with the authors ZQ and ZhZ to the handling editor at the time of review.

## Publisher's note

All claims expressed in this article are solely those of the authors and do not necessarily represent those of their affiliated organizations, or those of the publisher, the editors and the reviewers. Any product that may be evaluated in this article, or claim that may be made by its manufacturer, is not guaranteed or endorsed by the publisher.

## Abbreviations

BP: Bodily Pain
CACMS: China Academy of Chinese Medical Sciences
CP: Chronic pain
GH: General Health
HAMD: Hamilton Depression Rating Scale
HT: Health Transition
HAMA: Hamilton Anxiety Scale
MH: Mental Health
MADRS: Montgomery–Åsberg Depression Rating Scale
MDD: major depressive disorder
PF: Physical Functioning
PSQI: Pittsburgh Sleep Quality Index
RE: Role-Emotional
RP: Role-Physical
S: Social Functioning
SF-36: self-reported 36-Item Short Form Survey
SF-MPQ: self-reported McGill Pain Questionnaire
taVNS: transcutaneous auricular vagus nerve stimulation
VAS: visual analog scale
VT: Vitality.
